# Targeted mutation of EphB1 receptor prevents development of neuropathic hyperalgesia and physical dependence on morphine in mice

**DOI:** 10.1186/1744-8069-4-60

**Published:** 2008-11-21

**Authors:** Yuan Han, Xue-Song Song, Wen-Tao Liu, Mark Henkemeyer, Xue-Jun Song

**Affiliations:** 1 Jiangsu Province Key Laboratory of Anesthesiology and Center for Pain Research and Treatment, Xuzhou Medical College, Xuzhou, Jiangsu, PR China; 2 Department of Neurobiology, Parker University Research Institute, Dallas, Texas 75229, USA; 3 Department of Developmental Biology, University of Texas Southwestern Medical Center at Dallas, Dallas, Texas, USA

## Abstract

EphB receptor tyrosine kinases, which play important roles in synaptic connection and plasticity during development and in matured nervous system, have recently been implicated in processing of pain after nerve injury and morphine dependence. Subtypes of the EphB receptors that may contribute to the neuropathic pain and morphine dependence have not been identified. Here we demonstrate that the subtype EphB1 receptor is necessary for development of neuropathic pain and physical dependence on morphine. The results showed that peripheral nerve injury produced thermal hyperalgesia in wild-type (*EphB1+/+*) control littermate mice, but not in EphB1 receptor homozygous knockout (*EphB1-/-*) and heterozygous knockdown (*EphB1+/-*) mice. Hyperalgesia in the wild-type mice was inhibited by intrathecal administration of an EphB receptor blocking reagent EphB2-Fc (2 μg). Intrathecal administration of an EphB receptor activator ephrinB1-Fc (1 μg) evoked thermal hyperalgesia in *EphB1+/+*, but not *EphB1-/- *and *EphB1+/- *mice. Cellularly, nerve injury-induced hyperexcitability of the medium-sized dorsal root ganglion neurons was prevented in *EphB1-/- *and *EphB1+/- *mice. In chronically morphine-treated mice, most of the behavioral signs and the overall score of naloxone-precipitated withdrawal were largely diminished in *EphB1-/- *mice compared to those in the wild-type. These findings indicate that the EphB1 receptor is necessary for development of neuropathic pain and physical dependence on morphine and suggest that the EphB1 receptor is a potential target for preventing, minimizing, or reversing the development of neuropathic pain and opiate dependence.

## Background

There are striking similarities between neuropathic pain and opiate withdrawal-induced pain enhancement. Mechanisms of neuropathic pain and opiate dependence are complex and involve factors at the levels of receptors, ion channels, the cell and neural networks. Roles of diverse neurotransmitters, receptor systems and intracellular signaling proteins have been demonstrated in both neuropathic pain [1-12] and opiate dependence [13-24]. For instance, the system of glutamate/N-methyl-D-aspartate (NMDA) receptors/nitric oxide (NO) cascade is critically important to the development of neuropathic pain and morphine dependence and withdrawal [11,13,14,18,19,22-26]. However, the specific cellular and molecular mechanisms that control induction and maintenance of neuropathic pain and morphine dependence remain unclear. We have recently demonstrated a possibility that nerve injury or prolonged μ-opioid receptor (MOR) activation may elicit neuronal alterations that recapitulate events during development [27-29]. Certain molecules and the molecule-mediated activities that are important during development and "silent" in matured nervous system may become activated after nerve injury or prolonged MOR activation and therefore involve in development of neuropathic pain and opiate dependence.

Receptor tyrosine kinases (RTKs) play vital roles in transmitting external signals to the inside of many types of cells. Eph RTKs and ephrins are involved in tissue-border formation, cell migration, axon guidance, synapse formation and neural circuit assembly during development of the nervous system [30-33]. EphB receptors can also regulate development of glutamatergic synapses and their plasticity in adult nervous system by interaction with NMDA receptors [34-36]. The NMDA receptors have an established role in neural plasticity and are fundamental mediators of expression, development and maintenance of neuropathic pain and opiate dependence [21,37-40]. Activation of the NMDA receptors results in Ca^2+ ^influx through the NMDA receptor ion-channel complex. The subsequent activation of various Ca^2+^-dependent enzymes, such as Ca^2+^/calmodulin-dependent kinase (CaMK) [41-43] and extracellular signal-regulated kinase (ERK) [44] play a critical role in induction of neuropathic pain and/or persistent opioid effects [40]. EphB receptors continue to be expressed (at lower levels) in the adult nervous system and, after neural injury [28,29] or prolonged MOR activation [27]. They are upregulated and redistributed in neurons, reactive astrocytes and oligodendrocytes [27-29,45-50]. Recent studies have shown that the EphB receptors can modulate sensory neuron excitability and spinal synaptic plasticity in acute inflammatory pain [51], neuropathic pain [9,28,29,52] and opiate dependence [27]. These studies demonstrate a critical role of the EphB receptors in the development of neuropathic pain and morphine dependence. Because of unavailability of the reagents and antibodies that could selectively activate and/or block a subtype of EphB receptor family, the specific EphB receptor that may play a key role in neuropathic pain and/or opiate dependence has not been identified. This study, using the EphB1 receptor homozygous knockout (*EphB1-/-*) and heterozygous knockdown (*EphB1+/-*) mice, provides the first evidence that the EphB1 receptor is required for development of neuropathic hyperalgesia and morphine dependence.

## Results

### EphB2-Fc inhibits nerve injury-induced thermal hyperalgesia in WT mice

**Figure 1:**
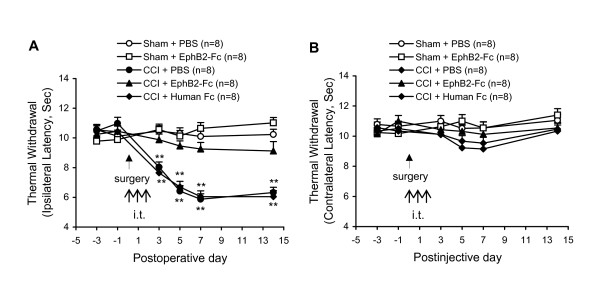
**An EphB receptor blocking reagent prevents thermal hyperalgesia after nerve injury.** Repeated measurements are shown of thermal sensitivity of the foot withdrawal response in CCI- and sham-operated mice injected with EphB receptors blocking reagent EphB2-Fc (each 2 μg for continuous 3 days) and control vehicles PBS or IgG-Fc (2 μg). The arrow(s) indicates surgery (sham or CCI) or drug injection (i.t.) at the time point. Data represent changes of the withdrawals of the ipsilateral foot (A) and the contralateral (B). The numbers of mice in each group are shown in parentheses. **P < 0.01 indicate significant differences between sham group (Sham + PBS) and each of the other groups.

We began by confirming and extending our earlier demonstrations in rats that multiple intrathecal administration (i.t.) of EphB receptor blocking reagent EphB1-Fc or EphB2-Fc can inhibit production of CCI-induced thermal hyperalgesia [29]. As shown in Fig. 1, repetitive daily injection of EphB2-Fc (2 μg, i.t.) for 3 days, starting 30 min prior to injury, significantly inhibited CCI-induced thermal hyperalgesia for at least 14 days, the last tested day (Fig. 1A). The slight increase in thermal sensitivity of the foot contralateral to CCI treatment on the postoperative 5^th ^and 7^th ^day (groups of CCI + PBS and CCI + IgG-Fc) also disappeared after EphB2-Fc treatment (Fig. 1B). The EphB2-Fc treatment did not significantly affect thermal sensitivity in the sham-operated animals. Injections of PBS or IgG-Fc (2 μg, i.t.) did not significantly alter thermal sensitivity in the CCI and sham-operated mice. These results obtained in mice are similar to those we have recently reported in rats [29].

### Nerve injury-induced thermal hyperalgesia are prevented or largely diminished in EphB1-/- and EphB1+/- mice

**Figure 2:**
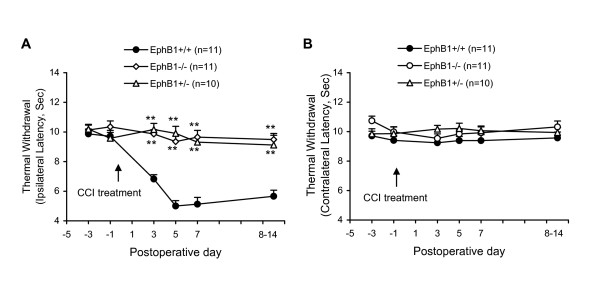
**Nerve injury-induced changes of thermal sensitivity in *EphB1-/-*, *EphB1+/- *and *EphB+/+ *mice.** The arrow indicates CCI treatment at the time point. Data represent changes of the hindpaw thermal withdrawals of the ipsilateral foot (A) and the contralateral (B). The numbers of mice in each group are shown in parentheses. **P < 0.01 indicates significant differences between groups of *EphB1-/- *and *EphB1+/+ *and groups of *EphB1+/- *and *EphB1+/+*.

Current available reagents, such as the ephrinB1-Fc, ephrinB2-Fc, EphB1-Fc and EphB2-Fc used in the present and previous studies [29,51], do not distinguish among different types of the ephrinBs and EphB receptors. Here we tested the role of the subtype EphB1 receptor in neuropathic pain using *EphB1-/- *and *EphB1+/- *mice. The results showed that CCI treatment induced thermal hyperalgesia in more than 90% of the WT, but in less than 10% of the *EphB1-/- *and *EphB1+/- *mice. Data are summarized in Table 1. Before receiving CCI treatment, the *EphB1-/- *and *EphB1+/- *and the WT mice exhibited similar thermal sensitivity. The time courses and changes of thermal sensitivity in *EphB1+/+*, *EphB1+/- *and *EphB1-/- *mice are shown in Fig. 2. The withdrawal latencies of the feet ipsilateral to CCI treatment in *EphB1+/+*, but not *EphB1-/- *and *EphB1+/- *mice were significantly decreased from the previous values each day after surgery, throughout the last test on the day 14 after surgery (Fig. 2A). Withdrawal latencies of the feet contralateral to CCI treatment in all of the three groups of mice were not significantly changed before and after surgery (Fig. 2B). Some of these mice were further used for electrophysiological recordings 8–14 days after nerve injury. In addition, 1/12 *EphB1-/- *and 1/11 *EphB1+/- *mice (see Table 1) showed thermal hyperalgesia after CCI treatment. The latencies of thermal withdrawal in these two mice decreased from preoperative values of 10–11 s to 7–8 s (~60%). Meanwhile, 1/12 WT mice that received CCI treatment showed no hyperalgesia. These three mice were not included in Fig. 2. We also observed the posture and gait and motor behavior related to the thermal test in those WT, *EphB1-/- *and *EphB1+/- *mice and did not find obvious differences between the WT and the *EphB1-/- *and EphB+/- mice.

**Table 1:** Thermal hyperalgesia in wide type and EphB1 receptor knockout or knockdown mice after nerve injury (CCI treatment) and intrathecal administration of ephrinB1-Fc.

Group	Total mice	Number of mice (%)	
		with hyperalgesia	without hyperalgesia
CCI treatment			
EphB1+/+	12	11 (91.7%)	1 (8.3%)
EphB1-/-	12	1 (8.3%)	11 (91.7%)
EphB1+/-	11	1 (9.1%)	10 (90.9%)
			
EphrinB1-Fc (1 μg, i.t)			
EphB1+/+	10	10 (100%)	0
EphB1-/-	10	0	10 (100%)
EphB1+/-	12	2 (16.67%)	10 (83.33%)

### EphrinB1-Fc evokes thermal hyperalgesia in EphB1+/+, but not EphB1-/- and EphB1+/- mice

**Figure 3:**
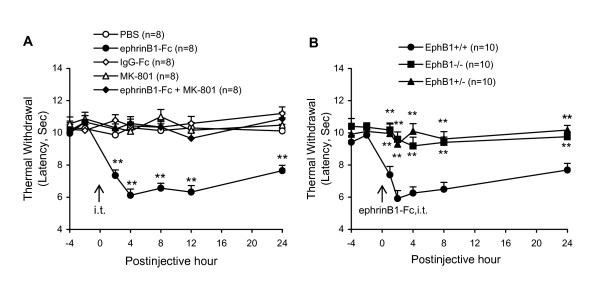
**EphrinB1-Fc-induced changes of thermal sensitivity in *EphB1-/-*, *EphB1+/- *and *EphB+/+ *mice.** A: EphrinB1-Fc (1 μg)-induced thermal hypersensitivity in WT mice is prevented by NMDA receptor antagonist MK-801 (5 μg, pretreated). Injection of IgG-Fc (1 μg) did not change the thermal sensitivity, nor did MK-801 alone affect the responses. B: EphrinB1-Fc (1 μg) induced thermal hypersensitivity in *EphB1+/+*, but not in *EphB1+/- *and *EphB1-/- *mice. The values of thermal withdrawal are mean values of both feet. The numbers of mice in each group are shown in parentheses. The arrow indicates drug injection (i.t.) at the time point. **P < 0.01 indicate significant differences between groups of the different treatments and the PBS control.

EphrinB1-Fc can activate EphB receptors and evoke thermal hyperalgesia via interaction with the NMDA receptor in rats [29,51]. The results here showed that a single injection of ephrinB1-Fc (1 μg, i.t.) caused a rapid onset (within 2 h) and prolonged (at least 24 h) thermal hypersensitivity in mice. EphrinB1-Fc-induced thermal hypersensitivity was prevented by a prior injection of an NMDA receptor antagonist, MK-801 (5 μg). IgG-Fc (2 μg, i.t.) did not produce thermal hypersensitivity. Data are summarized in Fig. 3A. These results in mice are similar to that in rats we have recently reported [29,51].

Additional experiments were performed in *EphB1+/- *and *EphB1-/- *mice to test if the EphB1 receptor is required for the ephrinB1-Fc-induced thermal hyperalgesia. The results showed that ephrinB1-Fc (1 μg, i.t.) produced thermal hyperalgesia in 100% of the *EphB1+/+ *mice tested, while in only 16.7% of the *EphB1+/- *and none (0%) of the *EphB1-/- *mice (Table 1). The time courses and changes of thermal sensitivity in *EphB1+/+*, *EphB1+/- *and *EphB1-/- *mice are shown in Fig. 3B. Data from the 16.7% (2/12) of *EphB1+/- *mice that exhibited moderate thermal hypersensitivity following ephrinB1-Fc injection were not included in Fig. 3.

### Nerve injury-induced DRG neuron hyperexcitability are prevented or largely diminished in EphB1-/- and EphB1+/- mice

Hyperexcitability of DRG neurons following nerve injury contributes to sensitization of the central nociceptive neurons in dorsal horn (DH) of the spinal cord, leading to chronic pain and hyperalgesia. Hyperexcitability of DRG neurons after CCI and other forms of injury is often manifested as depolarization of resting membrane potential (RMP), a decrease in action potential (AP) current threshold, and increased repetitive discharge [5,11,25,29,53-58]. We examined these three electrophysiological properties of the intact DRG neurons from WT and *EphB1-/- *and *EphB1+/- *mice to test the possibility that the EphB1 receptor contributes to DRG neuron hyperexcitability.

**Figure 4:**
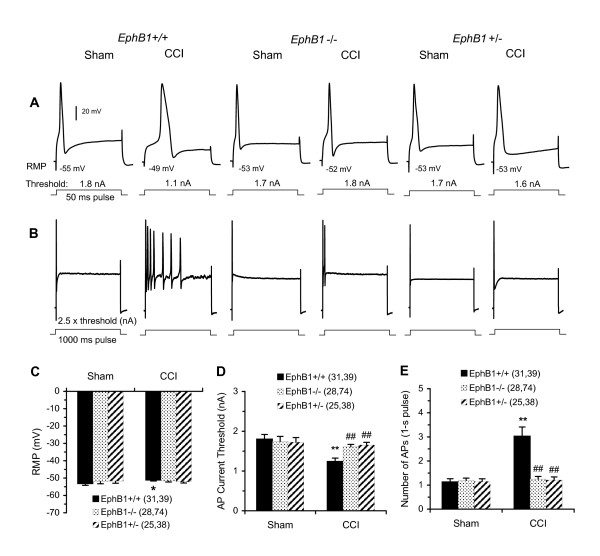
**Alteration of excitability of medium-sized DRG neurons from *EphB1-/-*, *EphB1+/- *and *EphB+/+ *mice.** A, Examples of neural responses recorded intracellularly during the test sequence used to determine AP threshold. Only one of the depolarizing 50-ms pulses (bottom) and corresponding responses (top) are illustrated in each case. B, Examples of neural discharge patterns evoked by depolarizing current. Alterations of the RMP, AP threshold current and repetitive discharge are summarized in C, D and E, respectively. The numbers of cells tested in each group are shown in parentheses. *P < 0.05, **P < 0.01 indicate significant differences compared with the group of sham *EphB1+/+*. ##P < 0.01 indicate the significant differences compared with the group of CCI-treated *EphB1+/+*.

A total of 235 medium-sized neurons [diameter (mean ± SE): 39.6 ± 0.43 μm] were recorded from the intact L_4 _and/or L_5 _DRG from the *EphB1+/+, EphB1+/- and EphB1-/- *mice that previously received sham or CCI treatment (from those shown in Fig. 2), respectively. The results showed that CCI treatment significantly increased excitability of the neurons from the WT mice, the RMP was depolarized, the AP current threshold lowered, the repetitive discharge following depolarizing current increased. However, CCI treatment did not significantly alter these properties of the DRG neurons from *EphB1+/- and EphB1-/- *mice. Examples are given and data summarized in Fig. 4. These results indicate that a proper level of EphB1 receptor is required for the development of hyperexcitability of DRG neurons after nerve injury in mice.

### Behavioral signs of naloxone-precipitated morphine withdrawal are largely diminished in EphB1-/- mice

**Figure 5:**
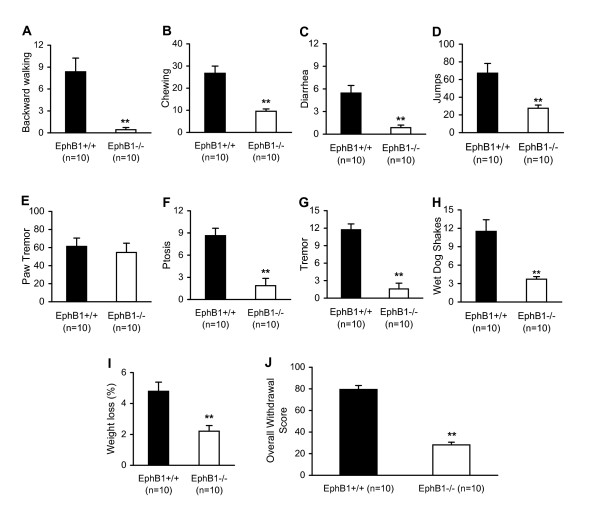
**EphB1 receptor knockout attenuates behavioral signs of morphine withdrawal.** A-I: Behavioral signs of naloxone-precipitated morphine withdrawal in *EphB1+/+ *(wild-type, WT) and *EphB1-/- *(EphB1 knockout) mice. Those mice were injected (i.p.) with repeated pulses of morphine given in 7 escalating doses every 8 h (20, 40, 60, 80, 100, 100, and 100 mg/kg). Two hours after the last morphine injection, mice were injected with naloxone (1 mg/kg, s.c.), and withdrawal symptoms were monitored for 30 min after naloxone administration. J: Overall withdrawal score. In addition to measuring individual withdrawal signs (A-I), an overall opiate withdrawal score was calculated as (no. of backward walking steps × 0.1) + (diarrhea × 2) + (no. of jumps × 0.1) + (paw tremor × 0.1) + (ptosis) + (tremor) + (% weight loss × 5) + (no. of wet-dog shakes) as described in the Methods. The numbers of mice in each group are shown in parentheses in A-J (the ten mice in each group of EphB1+/+ and EphB1-/- through A-J were the same mice). **, p < 0.01, indicate significant differences compared with the WT control group.

Naloxone-precipitated morphine withdrawal results in a characteristic morbidity, including anxiety, nausea, insomnia, hot and cold flashes, muscle aches, perspiration, diarrhea, etc. As shown in Figure 5, most of the observed behavioral signs, the backward walking, chewing, diarrhea, jump, ptosis, tremor, wet dog shake and weight loss were significantly alleviated in *EphB1-/- *mice compared to those in WT mice except that the paw tremor in *EphB1-/- *mice was not different from that in WT mice (Fig. 5I). Consistent with these changes, the overall withdrawal score significantly decreased in *EphB1-/- *mice compared to that in WT mice (Fig. 5J). These results suggest that the EphB1 receptor may be required for development of morphine dependence.

## Discussion

This study investigated roles of subtype EphB1 of EphB receptors in development of neuropathic pain and morphine dependence. The major findings are 1) peripheral nerve injury (CCI treatment) produces significant thermal hyperalgesia in WT, but not *EphB1-/- *and *EphB1+/- *mice. Meanwhile, CCI-induced thermal hyperalgesia in WT mice is inhibited by spinal administration of a reagent that can block the EphB receptors; 2) spinal administration of a reagent that can activate EphB receptors evokes thermal hyperalgesia in WT, but not *EphB1-/- *and *EphB1+/- *mice; 3) EphB1 receptor knockout or down prevents CCI-induced DRG neuron hyperexcitability; and 4) behavioral signs of morphine withdrawal are greatly suppressed in *EphB1-/- *mice compared to those in WT mice. These findings provide the first evidence that the subtype EphB1 receptor is necessary for development of thermal hyperalgesia and the associated sensory neuron hyperexcitability after nerve injury and pain enhancement following morphine withdrawal.

### EphB1 receptor is necessary for development of neuropathic hyperalgesia and morphine dependence

Recent studies have indicated that EphB receptors, which play an important role in synaptic connection and plasticity during development and in matured nervous system, are involved in pain processing [28,29,51,52] and opiate dependence [27]. We have recently shown that ephrinBs and EphB receptor proteins are upregulated and redistributed in DRG and DH after nerve injury [28,29], EphB receptor proteins upregulated in DH after chronic morphine treatment [27], and activation of EphB receptors is required for the development of neuropathic pain [28,29] and morphine dependence [27]. The Eph-receptors constitute the largest subfamily of RTKs in human genome, with 13 members divided into an A-subclass (EphA1-EphA8) and a B-subclass (EphB1-EphB4, EphB6) [59,60]. Although the importance of EphB receptors in neuropathic pain and morphine dependence has been demonstrated, the specific subtypes of EphB receptors have not been identified because of the unavailability of reagents and antibodies that could selectively activate and/or block an EphB receptor subtype. This study, using EphB1 receptor knockout (*EphB1-/-*) and down (*EphB1+/-*) mice, demonstrates, for the first time, that the subtype EphB1 receptor is required for the development of neuropathic hyperalgesia and morphine dependence. In addition, our results show that knockdown of the EphB1 receptor produces similar effect on thermal sensitivity as knockout. This result further suggests that a proper expression levels of the WT EphB1 receptor (*EphB1+/+*) is necessary for development of neuropathic hyperalgesia, and that reduction to approximately 50% normal levels in heterozygous mice sufficient to prevent this form occurring.

### The forward signaling of EphB1 receptor is involved in development of neuropathic hyperalgesia and morphine dependence

The ephrinB-EphB interaction is well known to lead bidirectional signals which are propagated into the ephrinB-expressing cells (reverse signaling) and the EphB-expressing cells (forward signaling). Depending upon context, both forward signaling and reverse signaling play important roles, either pre- or postsynaptically, in synaptic plasticity. Upregulation and redistribution of the EphB receptors in the DH after nerve injury [28,29] or prolonged MOR activation [27] may initiate a cascade of postsynaptic effects, starting with EphB receptor activation, then intracellular signaling through Src-family kinases[51,61], and resulting in NMDA receptor activation [1-4,6-12] with Ca^2+ ^influx through the NMDA receptor ion-channel complex. The NR2B subunit of NMDA receptor activation may be important for morphine dependence [27]. The subsequent activation of various Ca^2+^-dependent signaling pathways [38,62] plays a central role in neuropathic pain and morphine dependence. The Ca^2+^/CaMKII protein can phosphorylate (activate) cyclic AMP related element binding (CREB), which leads to increases in c-Fos mRNA and c-Fos protein expression [63]. Gene expression is thought to play an important role in many forms of plasticity, including hyperalgesia and morphine dependence. The present study further shows that binding to the EphB1 receptor is required for ephrinBs such as ephrinB1-Fc, which may also bind to EphB1-4 and EphA4 receptors, to promote behavioral hyperalgesia and that the EphB1 receptor protein-null mutant mice fail to development hyperalgesia or morphine dependence. These findings support a role of the EphB1 receptor forward signaling in neuropathic hyperalgesia and opiate dependence.

In addition, we have noticed that a small proportion of *EphB1-/- *and *EphB1+/- *mice (~10%) exhibit hyperalgesia after nerve injury or spinal administration of ephrinB1-Fc (see Table 1). Possible reasons may include that some pain related signals, the neurotransmitters, neuromodulators, receptors, ion channels and intracellular pathways that we have not examined, might have been modified or integrated in the *EphB1-/- *and *EphB1+/- *mice.

### Roles of the reverse signaling of EphB1 receptor in development of neuropathic hyperalgesia and morphine dependence

EphB stimulation of reverse signaling through activation of ephrinBs may also be involved in the development of neuropathic hyperalgesia after nerve injury. Upregulation and redistribution of ephrinBs in DRG and DH [28,29] may be initiated/regulated by the nerve injury, but probably also caused by increased reverse signaling mediated by EphB receptors in DRG and/or DH where EphB receptor proteins are upregulated after nerve injury [28,29]. If so, loss of postsynaptic EphB1 receptor in the EphB1-/- mice may result in loss of reverse signaling and therefore the subsequent activities. In contrast, chronic morphine treatment causes significant upregulation of EphB receptor protein in mice spinal cord, while expression of ephrinBs (ephrinB1, ephrinB2, and PY99) remains unchanged [27]. The behavioral and neurochemical signs of morphine dependence are largely diminished by the EphB receptor blocking reagent [27]. The present study further shows that the behavioral signs of morphine withdrawal are prevented in EphB1 receptor protein-null mice. Taken together, these findings suggest that reverse signaling may participate in development of neuropathic hyperalgesia, but seems to be less important in development of morphine dependence. The EphB1 receptor may act by interaction with the NR2B subtype of NMDA receptors [27] and then activate the postsynaptic signals ERKs [27,64] and CREB [27,65,66], or NO pathways [46,67], while the ephrinBs do not seem to be necessary in this process. However, at this point in our analysis there is no direct evidence that allows us to definitively rule in or out a role for reverse signaling in neuropathic hyperalgesia and morphine dependence.

### EphB1 receptor is necessary for development of sensory neuron hyperexcitability after nerve injury

Hyperexcitability of DRG neurons following nerve injury contributes to the sensitization of central nociceptive neurons in DH, leading to chronic pain and hyperalgesia. The findings that reagents that can activate the EphB receptors increase excitability and the reagents that can block the EphB receptors suppress hyperexcitability of the nerve-injured DRG neurons [29] suggest widespread effects of these receptors on the excitability of presynaptic primary sensory neurons and represent the first demonstration that ephrinB-EphB receptor signaling can regulate excitability in any neurons. This study provides further evidence that the EphB1 receptor knockout or down can prevent the DRG neurons from being hyper-excitable following nerve injury, suggesting that the EphB1 receptor is required for the development of DRG neuron hyperexcitability. These findings also indicate a sensory neuron mechanism underlying the behavioral analgesia in EphB1 receptor protein-null mutant mice. It remains unknown how the EphB receptors, which are upregulated in DRG and DH after nerve injury, regulate the DRG neuron excitability. One possibility is that activation of the EphB1 receptor may modulate the sodium channel Nav1.7 via regulation of the small G-protein Ras-ERK-mitogen activated protein kinase (MAPK) pathway [29,68-74]. The EphB receptors on DRG neurons would be activated directly by certain initial injury signals from peripheral and/or by the upregulated ephrinBs, by the reverse signaling of EphB receptors from DH through activation of ephrinBs, and probably by other retrograde signaling through ephrinBs. The signaling would be lost in the knockout or down mice DRG due to loss of the postsynaptic EphB1. There is lack of evidence supporting or excluding these possibilities.

### EphB1 receptor is a potential therapeutic target for neuropathic hyperalgesia and morphine dependence

Both neuropathic pain and opiate dependence continue to pose major challenges. Current drugs and nondrug therapies offer very limited substantial pain relief to patients. The present and several recent studies have provided evidence that the neuropathic hyperalgesia and morphine dependence can be markedly suppressed by knockout/down of EphB1 receptor and/or spinal administration of an EphB receptor blocking reagent or ephrinB2 siRNA [27-29,51,52]. These findings, in addition to supporting a novel mechanism contributing to neuropathic pain and opiate dependence and a novel role for EphB receptor signaling in development of neuropathic pain and morphine dependence, open up a new avenue to preventing, minimizing, or reversing the development of neuropathic pain and morphine dependence. The similar analgesic effect of EphB1 knockdown (*EphB1+/-*) to that of knockout (*EphB1-/-*) suggests a further implication in development of new analgesics.

## Conclusion

We here provide the first evidence that the subtype EphB1 of EphB receptors is necessary for the development of neuropathic pain and morphine dependence. We hypothesize that nerve injury and prolonged MOR activation can elicit neuronal alterations that may result in activation of certain molecules, such as the ephrinB-EphB receptor signaling, that are important in synaptic connection and plasticity during development and "silent" in matured nervous system. These molecules therefore become critical to the development of neuropathic pain or morphine dependence. The activated molecules may be the therapeutic targets for preventing, minimizing and/or treating neuropathic pain and physical dependence on opiate.

## Methods

### Animals

The generation of EphB1 receptor protein-null mutant mice has been previously described [75,76]. For the present study *EphB1+/- *heterozygous males and females in the CD-1 background were bred to obtain a cohort of homozygous knockout (*EphB1-/-*), heterozygous knockdown (*EphB1+/-*), and wild-type (WT) (*EphB1+/+*) control littermate adult mice (25–30 g-wt). All breeding was done by the Henkemeyer group and adult mice were provided to the Song group, who were blinded to the genotypes. Additional WT, male CD-1 mice (25–30 g-wt) (Fig. 1, 2) were obtained from Charles River Laboratories, MA, USA. All experimental procedures were conducted in accordance with the regulations of the ethics committee of the International Association for the Study of Pain and approved by Parker Research Institute Animal Care and Use Committee.

### Neuropathic pain model and assessment of thermal hyperalgesia

Neuropathic pain was produced by peripheral nerve injury mimicked by modification of model of chronic construction injury of the sciatic nerve (CCI) in rats [77]. In brief, the left common sciatic nerve of each mouse was exposed at the mid-thigh level. Proximal to the sciatic nerve's trifurcation, approximately 4 mm of nerve was freed of adhering tissue and three ligatures (6-0 chromic gut) were tied loosely around it with about 0.5 mm between ligatures. All surgeries were done under anesthesia induced by intraperitoneal injection (i.p.) of sodium pentobarbital (50 mg/kg). After surgery, the muscle and skin layers were sutured.

Thermal hyperalgesia was indicated by a decrease in the latency of foot withdrawal evoked by a radiant heat generated and controlled by a IITC Model 336 Analgesia Meter (Life Science, Series 8, Woodland Hill, CA). The protocol was similar to that we have described previously [5,11]. Each mouse was placed in a box (10 × 12 × 12 cm) containing a smooth, temperature-controlled glass floor. The heat source was focused on a portion of the hindpaw, which was flush against the glass, and a radiant thermal stimulus was delivered to that site. The stimulus shut off automatically when the hindpaw moved (or after 20 sec to prevent tissue damage). The intensity of the heat stimulus was maintained constant throughout all experiments. The elicited paw movement occurred at latency between 9 and 15 sec in control mice. Thermal stimuli were delivered 3 times to each hind paw at 5–10 min intervals. The change in latency of foot withdrawal to the heat stimulus was used to judge the thermal sensitivity. The mice were tested on each of two successive days prior to surgery. The withdrawal latencies of the feet ipsilateral and contralateral to nerve injury were expressed separately (Fig. 1, 2). For the results expressing i.t. ephrinB1-Fc-induced thermal hyperalgesia, the values are mean values of both feet (Fig. 3). To examine effects of multiple administrations of the drugs on CCI-induced thermal hyperalgesia, the postoperative tests were conducted 3, 5, 7 and on the day of electrophysiological recording (days 9–14). To test the effects of ephrinB1-Fc on the thermal sensitivity, the mice were tested on each of 2 successful hours prior to injection and the postinjective tests were conducted 2, 4, 8, 12 and 24 h after injection.

### Opiate Withdrawal

Mice were injected (i.p.) with repeated pulses of morphine (Sigma, St. Louis, MO) given in 7 escalating doses every 8 h (20, 40, 60, 80, 100, 100, and 100 mg/kg). Two hours after the last morphine injection, mice were injected with naloxone (Sigma) (1 mg/kg, s.c.), and withdrawal symptoms were monitored for 30 min after naloxone administration. In addition to measuring individual withdrawal signs, an overall opiate withdrawal score was calculated as (no. of backward walking steps × 0.1) + (diarrhea × 2) + (no. of jumps × 0.1) + (paw tremor × 0.1) + (ptosis) + (tremor) + (% weight loss × 5) + (no. of wet-dog shakes) [20].

### Excised, intact ganglion preparation

*In vitro *preparations of DRGs were made from L_4 _and/or L_5 _ganglia for electrophysiological studies from the WT, EphB1-/- and EphB1+/- mice. The procedure was similar to that described previously in rats [5,11]. Briefly, a laminectomy was performed under anesthesia after the last behavioral tests. Ice-cold, oxygenated, artificial cerebrospinal fluid (ACSF), consisting of (in mM) 130 NaCl, 3.5 KCl, 1.25 NaH_2_PO_4_, 24 NaHCO_3_, 10 dextrose, 1.2 MgCl_2_, and 1.2 CaCl_2 _(pH = 7.3) was dripped onto the surface of the ganglion during the surgical procedure. The ganglion was removed from the mouse and placed in a 35-mm petri dish filled with the ACSF. Under a dissecting microscope, the perineurium and epineurium were peeled away from the ganglion and the attached sciatic nerve and dorsal roots transected adjacent to the ganglion. The ganglion was then placed in the recording chamber and mounted on the stage of an upright microscope (BX50-WI, Olympus, Japan). A U-shaped stainless steel rod with 4 pieces of silver wire crossing from one side to the other was used to hold the ganglion gently in place. The DRG was incubated in the oxygenated ACSF at room temperature (21–22°C).

### Electrophysiological recordings

Intracellular recordings were made from the intact DRG somata 2–6 h after dissociation using conventional bridge-balance techniques (Axoclamp-2B, Axon Instruments, Foster City, CA) and analyzed with PCLAMP-8 under Windows 98 (Axon Instruments). Somata of the DRG neurons were classified visually by their diameters. Only the medium-sized (soma diameter 30–50 μm) DRG neurons were included in this study. Glass microelectrodes were fabricated with a Flaming/Brown micropipette puller (Model P-97/PC, Sutter Instruments.) and filled with 2 M potassium acetate (pH = 7.2). The protocols used to record and measure electrophysiological properties of the DRG neurons were similar to those we have described recently [5,11]. Resting membrane potential (RMP) was taken 2–3 min after RMP had stabilized. All neurons accepted for testing exhibited a stable RMP of -40 mV or more negative. Action potential (AP) current threshold was examined by delivering depolarizing currents of 0.05–4 nA (50 ms duration) in increments of 0.1–0.2 nA until an AP was evoked. Repetitive discharge of each neuron was measured by counting the spikes evoked by intracellular injection of standardized depolarizing currents at 2.5 × threshold strength (×1000 ms).

### Drug administration

For the experiments on CCI-induced hyperalgesia, the following vehicle and drugs were administrated intrathecally (i.t., all in 5 μl) by means of lumbar puncture under brief inhalational anesthesia: PBS, ephrinB1-Fc chimera (1 μg), EphB2-Fc chimera (2 μg), human IgG-Fc fragment (IgG-Fc, 2 μg) and MK-801 (5 μg). The PBS was used as vehicle control. The molecule ephrinB1-Fc chimera (mouse recombinant; E 0778, Sigma) was used as an EphB receptor activator and it can bind to EphB1-4 and EphA4. The molecule EphB2-Fc chimera (mouse recombinant; Sigma, E9402) was used as an EphB receptors blocking reagent and it can bind to ephrinB1-3. The IgG-Fc was used as Fc control (Jackson Laboratory, Bar Harbor, ME). MK-801 was used as an NMDA receptor antag onist.

### Statistical tests

SPSS Rel 15 (SPSS Inc., Chicago, IL) was used to conduct all the statistical analyses. Changes in withdrawal latencies over time were tested with two-way ANOVA with repeated measures followed by Bonferroni post hoc tests. One-way ANOVA followed by Bonferroni post hoc tests was used to test the hypothesis that RMP, excitability (including the AP current threshold and number of APs evoked by 1 s pulse) in CCI groups of EphB1+/+ mice were different from those of EphB1-/- and +/- and the sham WT groups. Individual t-tests were used to test specific hypotheses about differences between EphB1+/+ and EphB1-/- mice in behavioral responses to morphine withdrawal. Chi-square tests were used to identify differences in the incidence of effects. All data are presented as mean ± SE. Statistical results are considered significant if p < 0.05.

## Abbreviations

AP: action potential; CaMK: Ca^2+^/calmodulin-dependent kinase; CCI: Chronic constriction injury of the sciatic nerve; CREB: cyclic AMP related element binding; DRG: Dorsal root ganglion; DH: spinal dorsal horn; ERK: extracellular signal-regulated kinase; MAPK: mitogen activated protein kinase; MOR: μ-opioid receptor; NMDA: N-methyl-D-aspartate; NO: nitric oxide; RMP: resting membrane potential; RTK: receptor tyrosine kinase.

## Competing interests

The authors declare that they have no competing interests.

## Authors' contributions

XJS planned and participated in conducting the studies and wrote the paper. YH, XSS and WTL conducted the behavioral and/or electrophysiological studies and data analysis. MH generated and identified the EphB1 receptor protein-null mutant mice and kept stimulating discussion on the project with XJS. All the authors approved the final manuscript.
